# Dog Experts' Brains Distinguish Socially Relevant Body Postures Similarly in Dogs and Humans

**DOI:** 10.1371/journal.pone.0039145

**Published:** 2012-06-13

**Authors:** Miiamaaria V. Kujala, Jan Kujala, Synnöve Carlson, Riitta Hari

**Affiliations:** 1 Brain Research Unit, O.V. Lounasmaa Laboratory, Aalto University, Espoo, Finland; 2 Advanced Magnetic Imaging Centre, Aalto University, Espoo, Finland; 3 Neuroscience Unit, Institute of Biomedicine/Physiology, University of Helsinki, Helsinki, Finland; University of Bologna, Italy

## Abstract

We read conspecifics' social cues effortlessly, but little is known about our abilities to understand social gestures of other species. To investigate the neural underpinnings of such skills, we used functional magnetic resonance imaging to study the brain activity of experts and non-experts of dog behavior while they observed humans or dogs either interacting with, or facing away from a conspecific. The posterior superior temporal sulcus (pSTS) of both subject groups dissociated humans facing toward each other from humans facing away, and in dog experts, a distinction also occurred for dogs facing toward *vs.* away in a bilateral area extending from the pSTS to the inferior temporo-occipital cortex: the dissociation of dog behavior was significantly stronger in expert than control group. Furthermore, the control group had stronger pSTS responses to humans than dogs facing toward a conspecific, whereas in dog experts, the responses were of similar magnitude. These findings suggest that dog experts' brains distinguish socially relevant body postures similarly in dogs and humans.

## Introduction

Inspecting social interaction between two people from a third-person viewpoint engages brain areas supporting the analysis of human bodies, facial expressions, biological motion, and theory of mind [1], [2]. However, humans are not limited to understanding the behavior of other humans but can become expert interpreters of the gestural communication of other species, especially social mammals, such as domestic dogs. Even persons who have never owned a dog may recognize a dog's emotional state [3].

Earlier results propose that the brain mechanisms underlying human social cognition are utilized in the perception of non-human animals. For example, humans distinguish the direction of apparent movement from point-light walkers, irrespective of whether the walker represents a human or a non-human animal [4]. Moreover, the electrophysiological 140–170-ms face-sensitive brain responses, that are most prominent for human faces, are stronger to animal faces than to objects, such as tools or furniture [5]. Furthermore, when humans see a dog biting a piece of food, their motor mirroring network is activated in a similar manner as during viewing a comparable action of a human being [6]. Although the perception of non-human animals thus seems to be supported by similar neural mechanisms as the perception of conspecific human beings, the common and distinct aspects of processing social behavior of humans and non-human animals remain largely unknown.

**Figure 1:**
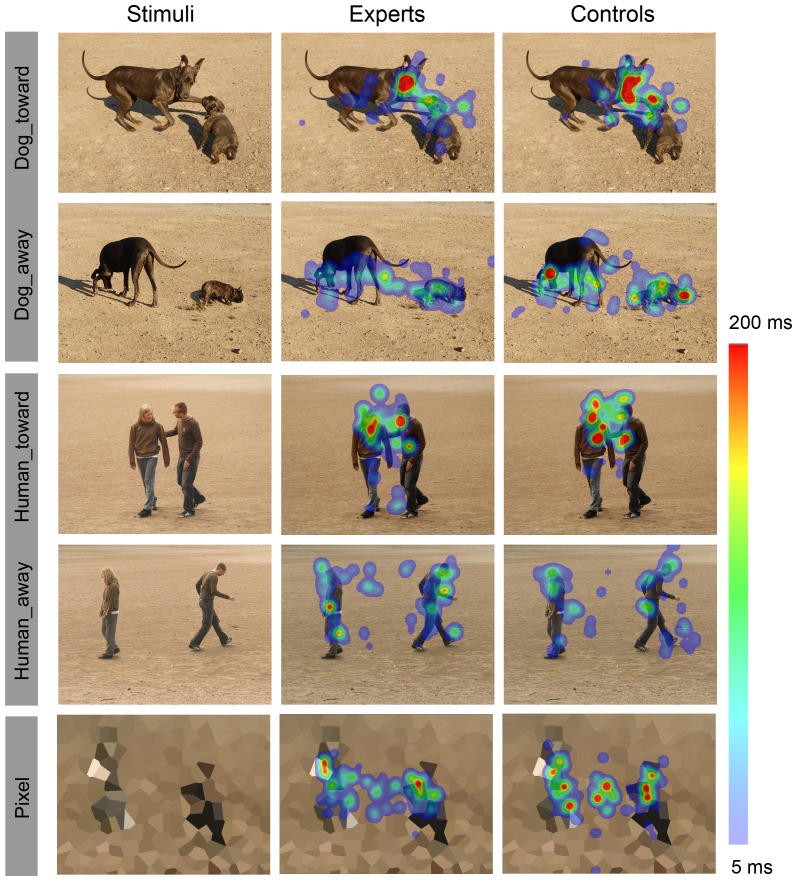
Examples of stimuli and eye gaze patterns. Left column: Examples of stimuli where dogs and humans were either engaged in face-to-face interaction (toward) or facing away from each other (away); the pixelated and crystallized versions of the human and dog photos (pixel) served as controls. Middle and right columns: The average eye gaze maps for experts and control subjects, respective to the stimuli on the left. The average fixation durations are color-coded (minimum of 5 ms indicated by light blue and the maximum of 200 ms or over by bright red).

Here, we investigated brain processes involved during observation of social interaction between two humans or two dogs. Since dog enthusiasts have a vast experience of observing dog behavior, we specifically tested whether such expertise would affect the observer's brain activity during observation of interaction of dogs. For this purpose, we measured functional magnetic resonance imaging (fMRI) signals from subjects observing photos of two humans or two dogs either interacting (facing towards each other) or not interacting (facing away); crystallized photos served as control stimuli (Figure 1).

Two subject groups participated in the study: *(i)* experts in dog behavior who were extensively involved in dog training and activities such as agility, obedience training, and game hunting, and *(ii)* a group of control subjects with no particular expertise with dogs. We expected that brain regions processing social interaction of humans would also process social interaction of dogs, at least in experts, and distinguish between the two interactive conditions (facing toward and facing away) for dogs as they do for humans [1].

## Results

### Expertise, empathy and mental state attribution

The background questionnaire quantified the group differences in expertise of dog behavior *(Ownership*, *Experience*, *Attachment and Knowledge)*. As expected, experts scored higher than the control group in all measures. The *Ownership* questionnaire confirmed that none of the control subjects owned a dog (only 4/18 control subjects had had a dog in the family earlier when they were children) whereas 17/19 dog experts owned at least one dog (difference statistically significant with Z = 2.7, *P*<0.001, two-sample Kolmogorov-Smirnov test). According to the *Experience* questionnaire, 8/19 experts and 0/18 control subjects had over 15 years of experience on dog behavior; 17/18 control subjects had zero experience (Z = 2.7, *P*<0.001). According to the *Attachment* questionnaire, 16/19 experts and 0/18 control subjects liked dogs extremely much (Z = 2.6, *P*<0.001), and the *Knowledge* questionnaire revealed that 14/19 experts but 0/18 control subjects had very much or extremely much knowledge of dog behavior (Z = 2.4, *P*<0.001).

The empathy scale (IRI) scores did not differ between expert and control groups in any subcategory (mean ± SD scores on *perspective taking* 17±4 and 17±5 for experts and controls, respectively, *P* = 0.7, multivariate GLM; *fantasy scale* 17±6 and 16±6, *P* = 0.7; *emotional concern* 18±5 and 17±5, *P* = 0.8; *personal distress* 10±4 and 9±3, *P* = 0.4).

The free written commentaries in the background questionnaire were answered by 89% of the dog experts and 67% of the control subjects. Dog experts made more inferences of dogs' mental states than did control subjects (18 and 5 inferences, respectively, Z = 3.1, *P* = 0.002, independent-samples Mann-Whitney U-test), whereas the number of mental state inferences of humans were equal (10 by experts and 8 by controls). No statistically significant differences were found in either group between mental state inferences of dogs and humans (18 dog and 10 human mental inferences by experts, T = 1.8, *P* = 0.072; 5 dog and 8 human inferences by control subjects, T = 1.0, *P* = 0.317, Wilcoxon signed ranks tests).

In general, the experts commented the dog photos in more detail, e.g. “*the poodle was a bit reserved*”, “*all dogs were female*”, and “*young*, *friendly*, *and playful dogs*”, whereas the control subjects commented them more generally by “*I couldn't always separate the poodle's head from the tail*”, “*dogs were often interested of one another*”, and “*dogs seemed to be in their natural surroundings*”. Both groups also declared having imagined figurines in the Pixel condition (5/19 dog experts; 6/18 control subjects).

### Eye gaze

Figure 1 shows examples of the eye gaze patterns. Depending on the condition, the subjects spent on average 977–1465 ms out of the total 2500-ms stimulus presentation time in viewing the dog and human ROIs (see Table 1). Due to technical artifacts (e.g. eye blinking), the sum of all fixation times per stimulus (including the background scenery) was 1672±41 ms (mean ± SEM across conditions and subjects).

**Table 1:** Mean ± SEM fixation durations (in ms) to dog and human stimulus ROIs and the background, averaged across subjects.

Stimulus condition	Part	Experts (N = 12)	Controls (N = 11)
Dog_toward	Head	878±123	1096±140
	Body	477±47	337±47
	Tail	35±7	32±8
	Head+body+tail	1390±95	1465±150
	Background	395±58	300±47
Dog_away	Head	451±68	597±82
	Body	619±37	534±41
	Tail	42±10	54±12
	Head+body+tail	1111±90	1184±81
	Background	494±51	471±53
Human_toward	Head	588±112	858±141
	Body	698±114	460±97
	Head+body+tail	1285±105	1318±114
	Background	439±56	461±92
Human_away	Head	311±58	473±111
	Body	666±118	549±108
	Head+body+tail	977±114	1022±108
	Background	479±73	577±83

Based on data from all stimulus pictures.

The total fixation durations to the heads and bodies of the creatures (both humans and dogs) did not differ between experts and control subjects (between-subjects factor *group*, F_1,21_ = 0.2, *P* = 0.7, repeated-measures ANOVA). Instead, a main effect of *behavior* was found (F_1,21_ = 67.8, *P*<0.001), as well as interaction effects between *species*×*body part* (F_1,21_ = 12.4, *P* = 0.002), *behavior*×*body part* (F_1,21_ = 40.7, *P*<0.001), and *species*×*behavior*×*body part* (F_1,21_ = 10.9, *P* = 0.003). The planned comparisons clarifying the ANOVA results showed that both human and dog heads received longer fixations in toward than away conditions (mean ± SEM fixation durations to dog heads were 462±56 ms longer in toward than away condition across groups, t_22_ = 8.3, *P*<0.001 and fixations to human heads were 329±54 ms longer in toward than away condition, t_22_ = 6.1; *P*<0.001; paired-samples t-tests), whereas the dog bodies received 168±38 ms longer fixations in away than toward condition (t_22_ = 4.5, *P*<0.001); fixations to human bodies did not differ between stimulus conditions. Furthermore, dog heads received 262±50 ms longer fixations than human heads in toward condition (t_22_ = 5.5, *P*<0.001) and 142±51 ms longer fixations in away condition (t_22_ = 2.7, *P* = 0.01), whereas the human bodies received 170±61 ms longer fixations than dog bodies in toward condition (t_22_ = 3.0, *P* = 0.007); fixation durations between human and dog bodies did not differ in the away condition.

The fixation durations to dog tails did not differ between groups nor conditions. However, the head/body ratios of fixation durations were smaller in experts than control subjects in both Dog_toward and Dog_away conditions (head/body ratio of experts 2.2±0.4 and controls 4.2±0.7 during Dog_toward: F_21_ = 5.3, *P* = 0.03, and head/body ratio of experts 0.7±0.1 and controls 1.2±0.2 during Dog_away: F_21_ = 4.8, *P* = 0.04, one-way ANOVA), whereas no statistical difference was reached for human stimuli (Human_toward: F_21_ = 2.4, *P* = 0.1; Human_away: F_21_ = 1.4, *P* = 0.3).

### Brain activations related to perception of dogs

**Figure 2:**
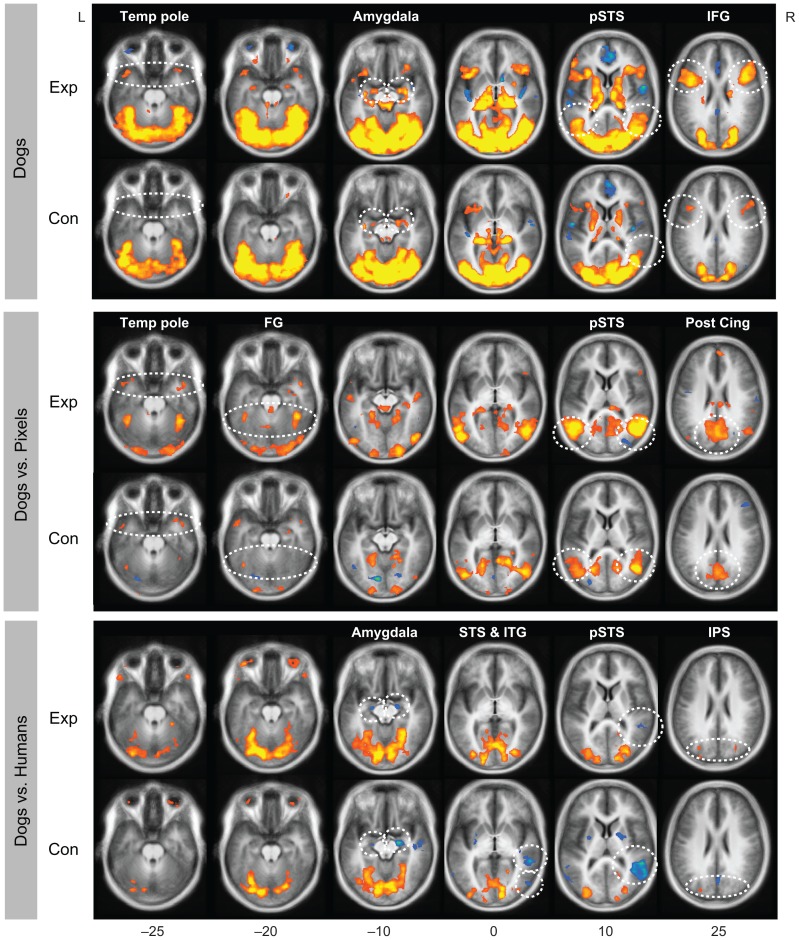
Brain activation of dog experts and control subjects in two-way statistical maps. Top panel: Dogs (Dog_toward+Dog_away). Middle panel: Activation to Dogs *vs.* Pixels. Bottom panel: Activation to Dogs *vs.* Humans. The FDR-corrected statistical maps (*q*<0.05) are overlaid on the anatomical MR image averaged across all subjects. Red-yellow: stronger activation in [first] *vs.* [second] stimulus category, blue-green: stronger activation in [second] *vs.* [first] category. Temp pole = temporal pole, pSTS = posterior superior temporal sulcus, IFG = inferior frontal gyrus, FG = fusiform gyrus, Post Cing = posterior cingulate, ITG = inferior temporal gyrus, IPS = intraparietal sulcus. Exp = experts, Con = controls.

The overall brain activations were very similar in both groups: dogs (“Dogs vs. Rest”; Figure 2 top panels) activated *e.g.* the occipital cortex, the temporal poles, amygdala, posterior superior temporal sulcus (pSTS), inferior frontal gyrus (IFG) and dorsomedial frontal cortex bilaterally. The activation map was very similar to the one acquired with the human stimuli in the contrast “Humans vs. Rest” in control subjects (see our previous report from the same subjects [1]). Pixel images activated other parts of this circuitry except the pSTS and amygdala.

The contrast “Dogs *vs.* Pixel” assessed physical differences between the stimuli and revealed stronger responses to dog than pixel images *e.g.* in the temporal poles, left fusiform gyrus (FG), bilateral pSTS conjoined with the temporoparietal junction, and posterior cingulate in both experts and control subjects (Figure 2, middle panels).

Both groups exhibited stronger activation to human than dog stimuli in the amygdala and the right pSTS, and control subjects also in the left pSTS and inferior temporal gyrus (ITG); the difference was most prominent in the right pSTS of the control subjects (see the blue-green color in the bottom section of Figure 2). In both groups, activation was stronger to dog than human stimuli in the intraparietal sulcus (IPS) bilaterally and in the early visual areas of the occipital cortex (Figure 2, bottom panels; orange-yellow).

### Activation of posterior temporal lobe linked to expertise on dog behavior

**Figure 3:**
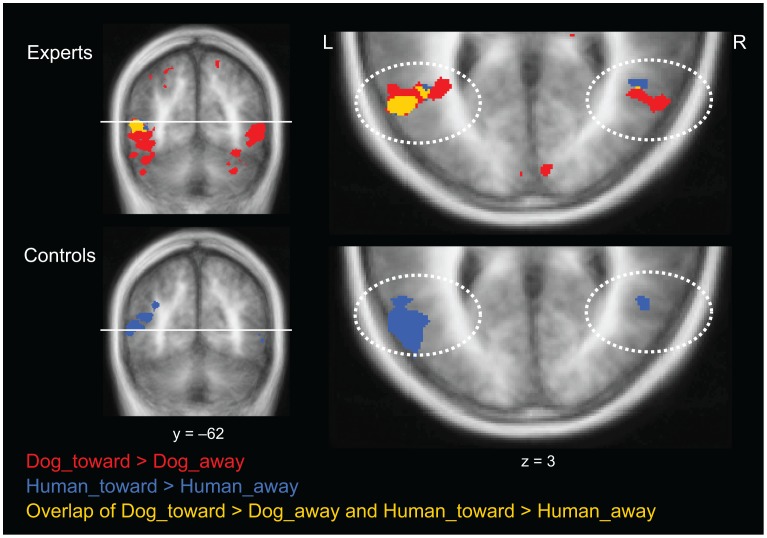
Distinction of interaction in experts and control subjects. Brain activation in pSTS in the contrasts Dog_toward>Dog_away (red) and Human_toward>Human_away (blue) and their overlap (yellow) in experts and control subjects. The location of the axial plane is indicated in the coronal image. All contrasts are shown at *q* (FDR)<0.05.

Brain processing influenced by the social interaction within the observed snapshot photos was inspected by contrasting conditions in which humans and dogs were facing toward a conspecific with the conditions in which they were facing away from a conspecific (Human_toward *vs.* Human_away and Dog_toward *vs.* Dog_away); see Figure 3 (top panel for experts and bottom panel for control subjects).

In both groups, the pSTS was activated more strongly in the Human_toward than Human_away condition (blue color in Figure 3). Stronger activity to Human_toward vs. Human_away condition was also seen in bilateral amygdala of both groups; for detailed discussion about the results of control subjects, see [1]. No statistically significant difference was detected in control subjects between Dog_toward and Dog_away conditions whereas in experts, several brain regions (frontal, parietal, insular and temporal regions) showed stronger activation to Dog_toward than Dog_away stimuli (see Table 2). In pSTS, the activation revealed by the Dog_toward>Dog_away contrast overlapped in both hemispheres with activation seen in the Human_toward>Human_away contrast (Figure 3 top).

**Table 2:** Brain activations of both groups for dogs facing toward each other vs. control conditions.

	Dog_toward>Dog_away				Dog_toward>Human_toward				
	Experts		Controls		Experts			Controls	
Brain area	mm^3^	Peak(x, y, z)	mm^3^	Peak(x, y, z)	mm^3^	Peak(x, y, z)		mm^3^	Peak(x, y, z)
Supramarginal gyrus (TPJ)	3952	59, −29, 30			270	−59, 28, 28			
Superior frontal gyrus	1476	−4, 49, 30							
	279	2, −5, 60							
	1258	−7, 55, 39							
	1343	−19, −20, 63							
	279	−4, 16, 57							
Middle frontal gyrus	273	−22, −14, 39			486	51, 17, 41			
Inferior frontal gyrus	1335	41, 16, 15			540	43, 35, 13			
	37786	−40, 37, 21							
Inferior frontal sulcus									
Cingulate gyrus	282	23, −47, 24							
	431	−1, 43, 6							
Insula	6319	47, −11, 6							
	1946	32, −14, 3							
Inferior temporal gyrus	8515^a^	44, −56, −3							
Precentral gyrus	1583	−46, −8, 39							
Superior parietal gyrus	297	23, −59, 60							
	11186	−58, −29, 27							
Intraparietal sulcus					567	43, −33, 44			
Superior occipital gyrus	270	−13, −95, 6							
Precuneus					297	−3, −70, 56			
Parieto-occipital sulcus					621	15, −52, 6			
Middle occipital gyrus	12535^b^	−28, −80, −18						2430	27,−84,5
								4752	−32, −81, 7
Calcarine sulcus	310	2, −89, 0						23949	−12,−80,−16^c^
Hippocampus	517	26, −29, 12			594	22, −30, 1			
	431	−16, −38, −3							
Lingual gyrus	349	−10, −86, −18			88452^c^	10, −76, −19			
	**Dog_away > Dog_toward**				**Human_toward > Dog_toward**				
Amygdala					324		18, −10, −12	1566	21,−12,−10
					675		−18, −16, −13		
pSTS								9342	46,−58,11
								756	−48,−64,6
								351	−65, −52, 5
Angular gyrus								1242	51,−66,28
Superior frontal gyrus								1242	6,59,37
Lingual gyrus	525	−7,−65,−3							

a) extends to middle occipital and fusiform gyrus.

b) extends to inferior temporal and fusiform gyrus and superior temporal sulcus.

c) extends to calcarine and intraparietal sulcus. All contrasts *q* (FDR)<0.05, k>10 voxels.

Additionally, the the scores of the expertise questionnaires were positively correlated with the magnitudes of brain activity within the pSTS during the “Dog_toward>Dog_away” contrast: all measures correlated statistically significantly with the right pSTS (*Ownership R* = 0.39, *P*<0.05; *Experience R* = 0.41, *P*<0.05; *Attachment R* = 0.41, *P*<0.01 and *Knowledge R* = 0.41, *P*<0.05) and Experience and Attachment measures correlated with the left pSTS (*Experience R* = 0.35, *P*<0.05; *Attachment R* = 0.39, *P*<0.05).

**Figure 4:**
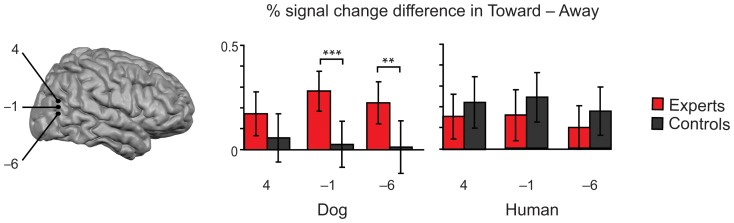
Difference between experts and control subjects in distinguishing body postures of dogs. Left: The locations of the three ROIs vertically adjacent to each other overlaid on the cortical surface. Right: Experts had stronger differences between Dog_toward – Dog_away conditions than control subjects at the two most ventral ROIs of right hemisphere (z = −1 and z = −6). There were no group differences in the signal change between Human_toward – Human_away conditions. ** p = 0.01, *** p<0.001.

Furthermore, the difference between Dog_toward *vs.* Dog_away conditions was stronger in experts than in the control group throughout the left hemisphere, as well as within the two most ventral ROIs of the right hemisphere [Figure 4; z coordinate = 4: F_1, 35_ = 9.749, *P* = 0.004 (left hemisphere); z = −1: F_1, 35_ = 5.994, *P* = 0.02 (left) and F_1, 35_ = 15.163, *P*<0.001 (right), z = −6: F_1, 35_ = 8.233, *P* = 0.007 (left) and F_1, 35_ = 7.048, *P* = 0.01 (right), one-way ANOVA].

Instead, the groups had equal response differences between Human_toward *vs.* Human_away conditions throughout vertically adjacent ROIs in both hemispheres (rightmost histogram in Figure 4).

### Sensitivity for conspecifics in amygdala?

**Figure 5:**
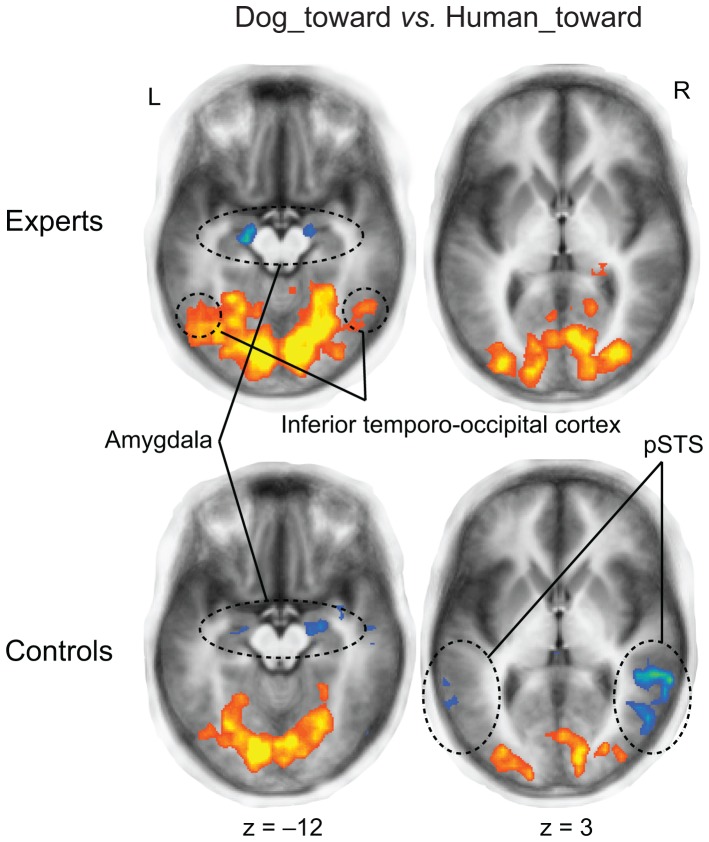
Brain activation to dogs vs. humans facing a conspecific. Differences in brain activation between Dog_toward and Human_toward at *q* (FDR)<0.05. Activation was stronger to Human_toward than Dog_toward in the amygdala of both groups (left panel), and in the pSTS of control subjects only (bottom right). Activation to Dog_toward was stronger than to Human_toward in the inferior temporo-occipital cortex of dog experts (top left). Smaller cluster size of k = 3 is used here to visualize the bilaterality of amygdala activation. Stronger activation to Dog_toward than Human_toward is depicted in blue color, and stronger activation to Human_toward than Dog_toward is depicted in red-yellow color.

In the Dog_toward vs. Human_toward contrast (Figure 5), the amygdala was activated less to Dog_toward than Human_toward in both experts and control subjects (depicted in blue in the leftmost slices of Figure 5), whereas the early visual areas were activated more strongly to Dog_toward than Human_toward (depicted in red–yellow in Figure 5). In dog experts, also the bilateral inferior temporo-occipital cortex (left upper corner in Figure 5), and right IFG, right IPS, right dorsal hippocampus, and right superior parietal gyrus were activated more strongly to Dog_toward than Human_toward stimuli (Table 2). Control subjects, but not experts, had weaker activation to Dog_toward than Human_toward stimuli also in the bilateral pSTS (depicted in blue in Figure 5).

## Discussion

### Observation of dogs in natural settings: the roles of mentalizing, visual perception and perspective-taking

In agreement with our expectations, the brain regions involved in processing of social interaction between humans also seemed to support observation of social interaction of dogs. The overall activation during observation of dogs (“Dogs vs. Rest”) was very similar in dog experts and control subjects, comprising areas that have been previously associated with socio-emotional processing, such as analysis of human bodies [7]–[12], biological motion [13]–[16], faces [17]–[19], gestures or expressions [20]–[22] and mentalizing [23]–[25]. The results suggest that generally, the social interaction of dogs is processed in the regions that also process human social cues, in both experts and non-experts of dog behavior.

Both dog experts and control subjects had stronger activation to dog than pixel images (“Dogs vs. Pixels”) in the temporal poles, pSTS, and posterior cingulate cortex, *i.e.* in brain areas that have been associated with various aspects of social cognition and mentalizing [23]–[25]. Although the subjects reported having made mental inferences, the dorsomedial prefrontal cortex (dmPFC), a part of the mentalizing circuitry (for review, see [26], [27]), showed no differential activity in the contrast “Dogs vs. Pixels” or “Dogs vs. Humans” in either group.

Previously, dmPFC activation has been often observed during tasks that require the subjects to make mental state inferences of persons whose gestures they do not see (e.g. [25], [28], [29]). However, visual perspective tasks, where subjects evaluated what other persons would see, have lacked dmPFC activation (e.g. [30], [31]), similarly as happened in the current study where the subjects had to interpret the observed bodily gestures in order to deduce the social interaction.

In one of the rare studies where subjects were observing two-person interactions, dmPFC activation differentiated “communicative intentions” from “private intentions” [32]. Furthermore, the dmPFC activation is found to be strengthened with the feeling of “personal presence” of the viewer to the social interaction [33]. We would like to emphasize that our basic contrasts “Dogs vs. Humans” and “Dogs vs. Pixels” did not differentiate between the interaction levels. However, the contrast Human_toward>Human_away revealed dmPFC activation in the control group, as reported earlier [1], and in the current study, the dog expert group (but not the control group), showed dmPFC activation in the “Dog_toward>Dog_away” contrast. These results agree with Walter and colleagues [32], since our “toward” conditions showed more communicative intention than ”away” conditions, suggesting that the dog communicative intentions were only detected by the dog expert group.

According to the IRI scores, the general empathy and perspective-taking abilities of our dog expert and control groups did not differ. Although the IRI samples empathy and perspective-taking toward humans instead of animals, one could have assumed that dog experts have over-developed perspective-taking abilities. Our results do not support this view, as the perspective-taking scores were not different between the groups. Altogether, our data thus suggest that expertise in dog behavior is not explained by differences in the mentalizing abilities between dog experts and control subjects. Instead, dog expertise seems to be more related to improved visual reading of the dogs' body postures. This interpretation is supported by the eye-tracking data that showed that the experts, compared with the control group, gazed relatively more the dog bodies than dog heads.

### Expertise affects the processing of dog interaction in pSTS and inferior temporo-occipital regions

The main aim of the present study was to investigate how the experience-derived expertise in dog behavior affects brain activation related to inspection of body postures reflecting social interaction between dogs. During observation of dog photos, activity in the frontal, temporal and parietal areas in the expert group, but not in the control subjects, differed between dogs facing toward and dogs facing away from each other. Furthermore, the lateral temporo-occipital cortex (pSTS region) was activated similarly in both groups to human photos but differently to dog photos. The dog experts' pSTS activation overlapped in the Human_toward>Human_away and in the Dog_toward>Dog_away contrasts, whereas in control subjects, the pSTS was activated only in the Human_toward>Human_away contrast.

The voxel cluster that showed a significant signal change in dog experts in the Dog_toward *vs.* Dog_away contrast continued from the pSTS (associated with biological motion [13]–[16] and social cues derived from eyes and bodies (for review, see [34])) ventrally to the inferior temporo-occipital regions associated with object processing (e.g. [9], [12], [35], [36]).

Recently, the pSTS has been shown to differentiate human motion from dog motion [37], but its activation is also modified by the level of human social interaction detected from visual cues [1], [2], [38]. Our data agree with these findings showing that the pSTS activation is modulated by the observed species (dog or human), the level of social interaction and, in addition, by the expertise of the subjects regarding the other species (dogs). In control subjects, the pSTS reacted stronger to interacting humans than to interacting dogs, without differences between dogs interacting and facing away. In the dog experts, on the other hand, activity in the pSTS did not differ between interacting humans and interacting dogs, but it differentiated between dogs interacting and facing away.

Additionally, the positive correlation between the pSTS activation of both hemispheres with the involvement of the subjects in dog behavior (the questionnaire factor *Experience*) and with their attachment to dogs (factor *Attachment*) suggest that the subjects' pSTS activation reflects the behavioral relevance of the social cues, even when they are seen from non-conspecifics.

Furthermore, the group comparison of signal changes within the ROIs in the temporo-occipital cortex showed that the signal difference between the Dog_toward and Dog_away conditions was stronger in experts than in control subjects in all ROIs of the left hemisphere and in the two most ventral ROIs of the right hemisphere. Thus also the lateral occipital cortex that is sensitive to object processing seems to be involved in the expertise of other species, especially in the right hemisphere. In fact, the common stimuli used in functional localization of the object-selective cortex include pictures of living creatures such as cats [39], dogs, or other animals [36], implying that besides inanimate objects, the area contributes to visual perception of animals. Furthermore, acquiring expertise in identifying objects increases response strength in the right lateral occipital gyrus and modifies the spatial distribution of activity [40]. Our results agree with the previous finding.

Earlier, expertise-related modulation of brain activity has been demonstrated in the fusiform gyrus in *e.g.* bird and car experts [18]. Although the focus of this study was not in this area, we did not find any differential FG processing between groups in the contrast Dogs *vs.* Humans.

### Eye gaze patterns during observation of social interaction

The total fixation durations to ROI regions (heads, bodies or tails) did not differ statistically significantly between experts and control subjects. In general, dog heads were fixated longer than human heads regardless of the condition, which may be due to the relatively bigger area of the stimuli covered by dog than human heads (mean 4704 vs. 2942 pixels, respectively). The total fixation time to both human and dog heads was longer in the toward than away conditions. The differences may be affected by the structure of the photos: in the toward conditions, the heads of both dogs and humans were close to each other–and close to the center of the photo–whereas in the away conditions, the distance between the heads was longer (as was the time between the fixations to the creatures). Alternatively, since gaze following is rather automatic (for a review, see [41]), gazes of humans/dogs in the toward condition guided the subject's gaze from one head to another, whereas in the away condition, the subjects spent more time following the gazes pointing elsewhere. These explanations also apply to longer fixation durations to dog bodies in the away than toward condition, since dog bodies were closer to each other in the away than toward condition and dog's gazes pointed away from each other.

Independently of the above low-level visual factors, the head/body ratio of fixation durations was smaller for experts than control subjects in both Dog_toward and Dog_away conditions, suggesting that the dog experts' gaze, compared with the control subjects' gaze, landed relatively longer to dog bodies than dog heads. The eye gaze is strongly task-dependent [42], and the results of the head/body ratio suggest that when the task was to inspect the attitudes of the interacting agents, experts in dog behavior spent a relatively longer time reading body than head postures of dogs compared with control subjects. The result is in line with previous data showing that humans who do not own pets, view dog faces with similar eye gaze patterns than human faces, i.e., by scanning mostly the eyes in both humans and non-human animals [43].

Importantly, the head/body ratio effect applied both to Dog_toward and Dog_away conditions, suggesting that the differences in brain activation observed in the pSTS and inferior temporo-occipital regions of experts between Dog_toward and Dog_away conditions were not due to differences in the fixation duration. However, the eye gaze data suggest that the experts were able to extract the social bodily gestures in both conditions better than control subjects, enabling them to distinguish the social situation.

### Human-sensitive responses in processing social interaction

The present study explored similarities and differences in neural processing of socially relevant body postures of humans and dogs. Both in experts and control subjects, observing photos of two interacting dogs elicited activation in the social brain circuitry in a very similar fashion than was previously shown for observing two interacting humans [1]. The direct contrasts of “Dogs vs. Humans” and “Dog_toward vs. Human_toward” also revealed some processing differences between the human and dog stimuli: visual cortex activation around the calcarine sulcus was, in both groups, stronger to dog than human pictures. This difference probably reflects the effect of slightly different filming conditions between these two types of stimuli: the background texture was more salient and contained more shadows in the dog than human stimuli.

The contrasts “Dogs vs. Humans” and “Dog_toward vs. Human_toward” also show stronger activations to humans than dogs in pSTS and amygdala. The pSTS activity was modified by expertise in dog behavior, but the activity in the amygdala was stronger for humans than dogs in both groups, regardless of the dog expertise. Activation in amygdala was stronger for humans than dogs in general (Dogs *vs.* Humans), stronger for interacting humans in Human_toward>Human_away contrast, and stronger for interacting humans than interacting dogs (Human_toward>Dog_toward). Our results are in line with the findings that amygdala activation differentiates faces of humans from those of dogs [44] and motion of humans from that of dogs [37]. The finding that the amygdala was activated only to human face-to-face interaction, but not to dog face-to-face interaction, suggests that the amygdala is sensitive to the human inter-personal distance viewed from the third-person perspective. The results of the current study further suggest that the amygdala is sensitive to inter-personal distance between other humans, but not between dogs.

## Materials and Methods

### Subjects

Altogether 42 healthy subjects participated in the measurements: 3 subjects in the pilot recordings, and 20 dog experts and 19 control subjects in the main study that comprised simultaneous fMRI and eye gaze measurements; subsequently, the subjects filled in behavioral questionnaires sampling empathy, exposure to dog behavior and mental state attribution (see details of the participants in Table 3). The study had a prior approval by the ethics committee of the Helsinki and Uusimaa district. All participants gave their written informed consent prior to the experiment, and a similar consent was also obtained from the actors before they were videotaped and photographed for stimulus production.

**Table 3:** Details of the participants in each of the measurements.

Measurement	Group	Subjects analyzed	Age/years (mean ± SD)	Females	Males
fMRI+Behavioral questionnaires	Dog experts	19	30.1±5.3	11	8
	Control subjects	18	28.3±6.8	9	9
Eye tracking	Dog experts	12	30.1±5.4	8	4
	Control subjects	11	27.0±7.3	4	7

Since experience plastically modifies neural function [45], expertise requirements in the study were based on active behavioral experience and exposure to dogs' behavior. Selection criteria of the dog expert group included long-lasting involvement in hobbies such as agility, dog obedience training, or game hunting, experience of dog behavior in dog-related jobs (veterinarians or dog-training teachers) in addition to currently owning one or more pet dogs. The participants of the control group did not own a dog, nor did they have extensive experience of dogs or a fear of them. Participants in both groups were selected after a short e-mail interview sampling the above requirements.

In the fMRI analysis, data from 2 female subjects were discarded due to excessive head motion, and thus both fMRI data and behavioral questionnaires were fully analyzed from 19 experts (11 females and 8 males, 18–39 years, mean ± SD 30.1±5.3 years) and 18 control subjects (9 females and 9 males, 19–41 years, mean ± SD 28.3±6.8 years). The subject age did not differ statistically significantly between the groups (t_36_ = 0.9, *P* = 0.4; two-tailed independent-samples t-test). Seventeen out of 19 experts and 17/18 control subjects were right-handed according to the Edinburgh Handedness Inventory [46]: on the scale from −1 (left) to +1 (right), the mean ± SD score for the expert group was 0.71±0.46 (range −1 to 1) and for the control group 0.77±0.41 (range −0.9 to 1). Subjects were compensated monetarily for the lost working hours and travel expenses.

Successful eye gaze recordings were obtained from 12 dog experts (8 females, 4 males, 18–43 years, mean ± SD 30.1±5.4 years) and 11 control subjects (4 females, 7 males, 19–41 years, mean ± SD 27.0±7.3). The subject ages did not differ between the groups (t_21_ = 1.9, *P* = 0.06; independent-samples t-test).

### Behavioral questionnaires

After the fMRI acquisition, the subjects filled in a background questionnaire concerning their expertise of dog behavior, with answers distributed to a five-point scale from 0 (min) to 4 (max), and an empathy questionnaire (Interpersonal Reactivity Index, IRI by [47]). The background questionnaire for expertise sampled the following features: *(i) Ownership*: How many dogs the subject currently owns (0 = none, 1 = one dog, 2 = two to three dogs, 3 = four to five dogs, 4 = more than five dogs), *(ii) Experience*: How many years the subject has been actively involved in dog training or hobby activities, e.g. agility or game hunting (0 = none, 1 = 1–5 yrs, 2 = 6–10 yrs, 3 = 11–15 yrs, 4 = over 15 years), *(iii) Attachment*: How much the subject likes dogs (0 = not at all, 1 = a little, 2 = some, 3 = very much, 4 = extremely much, *(iv) Knowledge*: How much the subject knows about dog behavior (0 = nothing, 1 = a little, 2 = some, 3 = very much, 4 = extremely much).

The questionnaire also included free space for elaborating the subject's dog- training expertise or other involvement in dog breeding, agility sports, obedience training, or equivalent. Moreover, the subjects were encouraged to write free commentaries on stimulus photos or the experiment to reveal their attention and reasoning during the scan. To check whether participants inferred the mental states of the individuals, one of the experimenters reviewed the free recall answers for descriptions of mental states or social references (“happy”, “playful”, “acting like they don't notice the other”, “were getting along well”), and tested them with the nonparametric independent-samples Mann-Whitney U-tests and Wilcoxon signed ranks tests.

Multivariate general linear model (GLM) was used to reveal possible statistically significant differences in empathy subscales between experts and control subjects, as well as between males and females, and the statistical significance of differences between experts and control subjects in the questionnaire scores was tested with the nonparametric Kolmogorov-Smirnov test.

### Stimuli

#### Preparation

For stimulus materials, students from the Theatre Academy of Finland and dogs and their owners from the local dog club (Espoon koirakerho) were recruited as “actors” in two separate filming sessions in the sand fields of nearby recreational parks. Both humans and dogs were filmed and photographed individually while they were inspecting their surroundings, and together with a conspecific while they were either ignoring or interacting with one another. This procedure resulted in 3810 still photos.

Ten photos from 4 actor pairs and 4 dog pairs were selected in both conditions, resulting in 40 photos per category. The pictures fulfilled the following criteria: *(i)* the photo was technically in focus and clearly presented the event in question (e.g. interaction between actors), *(ii)* the photos of the same category showed similar body orientations of the actors of the two species, *(iii)* a variety of different postures were captured for each actor (i.e. the different photos of one actor did not appear identical). Actors in the human stimuli were 2 males and 2 females, and the dogs in the dog stimuli were a French bulldog, a great dane, a cocker spaniel, and a poodle.

Some digital manipulation (e.g. removing human owners and the leash from a few dog photos) was done using Adobe® Photoshop® (version 7.0). The color schemes of the photos were equalized using Adobe® Photoshop® Lightroom® (version 1.3). Finally, a random sample of 40 stimulus photos from all human and dog categories were pixelated and crystallized (25 of them turned upside down to make identification of figures harder) to create a visual control category of pixel photos showing no social communication nor complete objects, yet consisting of separate observable shapes (see Figure 1 for examples). The rationale for the pixel category was to capture visual activation elicited by the gross contours and colors present within the human and dog photos, as well as to reveal task-related attempts to decipher social interaction.

This process resulted in 200 photos, comprising 5 categories of 40 images in each. The images were of 4 different actors per species, each appearing in 20 stimuli per category: *1*) two humans facing each other and greeting by shaking hands, hugging, or touching each other on the shoulder (*Human_toward*), *2*) two humans in the same photo but facing away and not noticing each other (*Human_away*), *3*) two dogs facing each other and greeting by sniffing and playing (*Dog_toward*), *4*) two dogs in the same photo but facing away (*Dog_away*), and *5*) crystallized pixel figures (*Pixel*) as control stimuli.

#### Presentation

The images, displayed on a projection screen by a data projector (Christie Vista ×3, Christie Digital Systems Inc., USA), were 640×480 pixels in size (width×height, 20 cm×14 cm on the screen), overlaid on a gray background of 1024×768 pixels and presented with a frame rate of 75 Hz. Stimulus presentation was controlled with Presentation® software (http://nbs.neuro-bs.com/) run on a PC computer.

The stimuli were viewed binocularly at a distance of 34 cm within a block design. Each stimulus was shown for 2.5 s in a continuous 25-s stimulus block, 10 stimuli per block, and the block alternated with 25-s rest blocks with a fixation cross on a grey background. The recording sessions started and ended with rest blocks, so that each session comprised 14 stimulus blocks and 15 rest blocks. The stimulus sequence also included blocks of photos of single humans and dogs in the same surroundings to establish a baseline of visual object perception. However, to emphasize the results between stimulus classes of “toward” and “away” categories, the single human and dog photos were excluded from the present study. The data were gathered in two successive recording sessions (each 12 min 5 s in duration, with 140 different stimuli presented in a pseudorandomized order). The order of the sessions was counterbalanced across subjects.

### Subject instruction

Prior to the experiment, the subjects were informed that they would see images of people and dogs, as well as abstract pixel compositions. They were instructed to explore the images freely and inspect the attitude of the beings towards one another or towards their surroundings, whenever possible. They were also asked to avoid overt and covert verbalizing and to keep the head still.

### Data acquisition

The magnetic resonance data were acquired with whole-body General Electric Signa® 3.0T MRI scanner at the Advanced Magnetic Imaging Centre. During the experiment, the subject was resting in the scanner, facing upwards and viewing the stimulus images through a mirror attached to the 8-channel head coil.

Functional MR images were acquired using a gradient-echo planar imaging sequence with field of view = 240×240 mm^2^, time of repetition = 2500 ms, time to echo = 32 ms, number of excitations = 1, flip angle = 75°, and matrix size = 64×64. Before the presentation of the visual stimuli, six dummy volumes were acquired allowing the MR signal to stabilize. Altogether 42 slices (thickness 3.0 mm) were acquired in an interleaved order. The resulting functional voxels were 3.75×3.75×3 mm^3^ in size.

Structural T1-weighted images were acquired using a spoiled-gradient echo sequence with a matrix size of 256×256, time of repetition 9.2 ms, field of view 260×260 mm^2^, flip angle of 15°, and slice thickness of 1 mm, resulting in 1×1.016×1.016 mm^3^ voxels.

### Data preprosessing

The fMRI data were analyzed with BrainVoyager QX software version 1.10.2/3 (Brain Innovation B.V., The Netherlands). Preprocessing included slice scan time correction and 3D motion correction with first volume as a reference, linear trend removal and high-pass filtering at 0.008 Hz.

Functional and anatomical data were iso-voxelated to 3×3×3 mm^3^ and 1×1×1 mm^3^ voxels, respectively, and normalized to the Talairach space [48]. All data were analyzed further at this resolution. Subsequently, all functional data were interpolated to the resolution of anatomical images for visualization purposes of the statistical maps.

### Statistical analysis of fMRI

Whole-brain analysis was conducted for identification of the activation differences during different stimulus conditions, separately for expert and control groups. Brain activations were subjected to statistical analysis using random effects general linear model (RFX-GLM), and the individual time courses were normalized using z-transformation. The predictors for RFX-GLM were obtained by convolving the time courses of the stimulus blocks with a canonical hemodynamic response function to reveal blood-oxygenation-level-dependent (BOLD) activations.

Main effects of dog stimuli were inspected with a bi-directional statistical map, showing contrasts in both directions within the whole brain, of “Dogs *vs.* Rest” (the term “Rest” referring here to the “baseline” level for the signal change; the Rest blocks were not modeled separately). Similarly, the object-related brain activations were inspected with a contrast of “Dogs *vs.* Pixel” (balanced for the amount of stimuli). The effect of species on brain activations was inspected with the bi-directional contrast “Dogs *vs.* Humans”. Thereafter, comparisons were made between humans facing toward each other and facing away (“Human_toward *vs.* Human_away”), dogs facing toward and away (“Dog_toward *vs.* Dog_away”), and humans and dogs in face-to-face interaction with a conspecific (“Human_toward *vs.* Dog_toward”). The relationship between the pSTS activation and the results of the expertise questionnaire in the “Dog_toward vs. Dog_away” contrast was further clarified by extracting the beta values from this region and calculating a Pearson correlation with each of the different expertise measures (*Ownership*, *Experience*, *Attachment and Knowledge*) across all subjects.

All statistical maps were corrected for multiple comparisons according to false discovery rate (FDR, [49]) incorporated in the BVQX software, and using a cluster size of 10 contiguous voxels [50]. The FDR-corrected statistical threshold was *q*(FDR)<0.005 for Dogs vs. Rest and *q*(FDR)<0.05 for the contrasts Dogs *vs.* Pixels, Dogs *vs.* Humans, Dog_toward *vs.* Dog_away, Dog_toward *vs.* Human_toward and Human_toward vs. Human_away. Brain areas showing modulation of activity were identified with common brain atlases [48], [51].

Since we were specifically interested in the expertise effects within the temporal-lobe areas responsive for perception of body postures and biological motion [8]–[10], [13], [14], [52]–[54], we defined anatomically three small ROIs of 8 mm^3^ in each hemisphere from the across-subjects MR images within the region important for perception of body postures and biological motion (Talairach coordinates of x = −52 and 52; y = −60; and z = 4, −1 and −6). The signal changes were examined for group differences with one-way ANOVAs in both hemispheres.

### Eye tracking

The subjects' eye gaze was tracked with SMI MEye Track long-range eye tracking system (Sensomotoric Instruments GmbH, Germany) to compare the number and duration of fixations around human and dog bodies in experts and control subjects. The tracking method is based on video-oculography and dark pupil–corneal reflection.

The infrared camera was set at the foot of the bed to monitor the subject's eye via a mirror attached to the head coil, and an infrared light source was placed on the mirror box to illuminate the eye. The camera was shielded properly (in house) and did not affect the signal-to-noise ratio of the fMRI data. The eye tracker was calibrated prior to the experiment using 5 fixation points, and the data were collected at a sampling rate of 60 Hz.

The eye gaze patterns (successful recordings obtained for 11 control subjects and 12 dog experts) were analyzed with Begaze 2.0 (Sensomotoric Instruments GmbH, Germany). Eye blinks were removed from the data and fixations were detected with a dispersion-threshold identification algorithm, using a 2° dispersion window and 120 ms as the minimum fixation duration. Gaze maps were then calculated separately for individual stimuli, by overlying the fixations of all subjects and by smoothing the data with a gaussian kernel of 70 pixels. Thereafter the average fixation duration was computed across subjects at each pixel and was color-coded for average fixation durations from 5 ms to 200 ms or more (Figure 1).

Eye movements between experts and control subjects were compared from all Human_toward, Human_away, Dog_toward and Dog_away stimuli (40 photos per category). For each photo, regions of interest were drawn manually around human heads and bodies as well as dog heads, bodies and tails and the remaining background image area, for which the subjects' total fixation durations were calculated.

The total fixation durations of experts and control subjects in each ROI were compared with a repeated-measures analysis of variance (ANOVA) with a between-subjects factor “*group*” (experts, controls) and within-subject factors “*species*” (human, dog), “*behavior*” (toward, away) and “*body part*” (head, body). The ANOVA results were clarified with planned contrasts (paired-samples t-tests) between dog and human body parts and interaction levels. Fixations to dog tails were inspected in a separate ANOVA with a between-subjects factor “*group*” and a within-subjects factor “*behavior*”. In addition, the ratios in fixation durations between heads and bodies were calculated and compared in one-way ANOVA with a between-subjects factor “*group*” and head/body ratios of all four stimulus categories.
